# Augmented repair of acute tendo Achilles ruptures with gastrosoleus turn down flap

**DOI:** 10.4103/0019-5413.73654

**Published:** 2011

**Authors:** Murat Demirel, Egemen Turhan, Ferit Dereboy, Tarik Yazar

**Affiliations:** Orthopaedic Surgeon, Bayindir Private Hospital, Ankara, Turkey; 1 Department of Orthopaedics, Zonguldak Karaelmas University, School of Medicine, Zonguldak, Turkey; 2 Orthopaedic Surgeon, Magnet Medical Center, Ankara, Turkey; 3 Department of Orthopaedics and Traumatology, Dr. Ankara University Faculty of Medicine, Ankara, Turkey

**Keywords:** Tendo Achilles, rupture, augmented repair, gastrosoleus turn down flap

## Abstract

**Background:**

We present the results of primary repair of acute tendo Achilles (TA) rupture augmented with gastrosoleus turn down flap technique.

**Patients and Methods:**

78 consecutive patients with a complete acute rupture of the Achilles tendon operated between 1993 and 2004 were included in study. We performed a modification of the Lindholm technique in which the primary Kessler suture repair of the tendon was augmented by a turn-down ~3 cm × 10 cm gastrosoleus aponeurosis flap. In all cases, a short-leg circular walking cast was applied at 90° of the ankle dorsiflexion for 3 weeks and all the patients were encouraged to full weightbearing ambulation immediately. After removal of the cast, isometric and isokinetic ankle exercises were performed for 3 weeks. Modified Rupp Score was used to evaluate the subjective satisfaction.

**Results:**

All of patients returned to daily activity and 54 (69%) of them returned to previous sport activity. The tendon repair failed in two patients and they were reoperated with an allograft. Three patients developed infection and one of them required débridement. One developed deep venous thrombosis and two permanent sural nerve injuries were encountered. One of the patients had a severe skin necrosis, which was treated with rotation flap. The mean Rupp score was 29 (3–33).

**Conclusion:**

Primary repair of acute tendo Achilles rupture augment with gastrosoleus turn down flip technique in combination of immediate weightbearing ambulation provides a good outcome, but is associated with similar complication rates to the previous literature.

## INTRODUCTION

Since first described by Ambroise Paré in 1575 and reported in the literature in 1633, rupture of the Achilles tendon has received increasing attention regarding treatment.^1^ As Achilles tendon rupture is both a serious injury and one of the most common tendinous lesions, this increasing attention naturally generates various treatment procedures.^2^,^3^

The treatment of Achilles tendon rupture includes conservative management and surgical intervention, but surgical repair seems to have been the preferred treatment in the late 1980s and 1990s.^4^–^7^ Non-operative treatment begins with an initial period of immobilization of the ankle in plantar flexion for 6 and 8 weeks using plaster cast or splints.^8^ Although non-operative treatment avoids the risk of surgery and decreases patient cost, this may result in a lengthened tendon with reduced power of the gastrosoleus muscle^9^,^10^ and a high rerupture rate.^4^,^6^

The main advantages of open repair are that it lowers the rerupturing rate of 2–5% and is convenient for patients who demand a short rehabilitation time due to work or sports requirements.^11^ Augmentation of end-to-end repair has been recommended for acute injury by many authorities, with fascia flaps or adjacent tendon.^7^,^12^–^14^ Lindholm *et al*. described the method of augmentation that reinforces the repair and prevents adhesion of the repaired tendon to the overlying skin.^12^ The two-sided gastrosoleus fascial flaps are rotated 180° and then twisted and sutured additionally to direct repair so that the smooth external surface faces the subcutaneous tissue. At our center, most of the acute ruptures of Achilles tendon are treated with this modified Lindholm technique. The purpose of this study was to report the results and complication of our case series of acute ruptures of the Achilles tendon using this technique.

## PATIENTS AND METHODS

Between April 1993 and February 2004, we consecutively treated 91 patients who had sustained a complete rupture of the TA with the modified Lindholm technique. This study was performed between the years 2002 and 2006. The inclusion criteria were acute ruptures, age over 18 years, unilateral rupture of the Achilles tendon and pre-injury ability to run, indicating functional level. Exclusion criteria were previous tendon rupture, previous tendon surgery and multiple injuries requiring delayed rehabilitation. Thirteen patients were lost to follow-up and were therefore excluded from the study. Thus we assessed 78 patients, 69 men and nine women with, mean age at the time of injury of 34 (26–48) years, with 44 right-sided and 34 left-sided injury. The dominant leg was involved in 51 patients and the non-dominant leg in 27 patients. Mean follow-up time was 4.6 (2–9) years. Surgery was performed on average 14.4 (0–4 days) hours from the injury.

Seventy-one patients (91%) had sustained the rupture during a sports-related activity. The modes of the injuries were football (*n*=46), basketball (*n*=16), tennis-squash (*n*=9) and a fall (*n*=7). Thirty-nine patients injured the Achilles tendon while playing football on a synthetic grass field. None of the patients had a history of tendonitis of the Achilles tendon or had been previously treated for a rupture on the opposite side. One of the patients had history of steroid inhalation and two patients had diabetes mellitus. The athletic level of 14 patients was competitive while of 57 patients was recreational [Table 1].

**Table 1:** Mode of injury

	Sports level		
	Competitive	Recreational	NA*
Football (*n*=46)	9	37	
Basketball (*n*=16)	3	13	
Tennis (*n*=7)	2	5	
Squash (*n*=2)		2	
Not during sports (*n*=7)			7
Total (*n*=78)	14	57	7

* NA: Non-athletic

Rupture of the Achilles tendon was diagnosed clinically by palpation of the defect and a positive calf-squeeze test. Anterior–posterior and lateral radiographs were made in order to exclude the possibility of associated avulsion fractures. No further ancillary testing other than physical examination and X-ray was utilized to verify an Achilles tendon rupture before the surgery.

### Operative technique

The tendon was repaired under regional (*n*=46) or general anesthesia (*n*=32) within mean of 14.4 hours (range 0–4 days) of injury. After tourniquet application to the thigh, patients were placed prone on the operating table and the ruptured tendon was approached through a medial incision parallel to the medial border of the Achilles tendon. The peritenon was then incised in the midline to avoid scarring of the peritenon to the skin incision. The sural nerve was left included in the lateral flap. During the operation, it was observed that all patients had a complete midsubstance tear of the Achilles tendon, 2–6 cm proximal to its insertion on the calcaneus.

The ends of the tendon were lightly débrided and reapproximated with a Kessler stitch of No. 2 Ethibond^®^ non-absorbable suture. A running circumferential suture with 3–0 Vicryl augmented the core suture.

**Figure 1:**
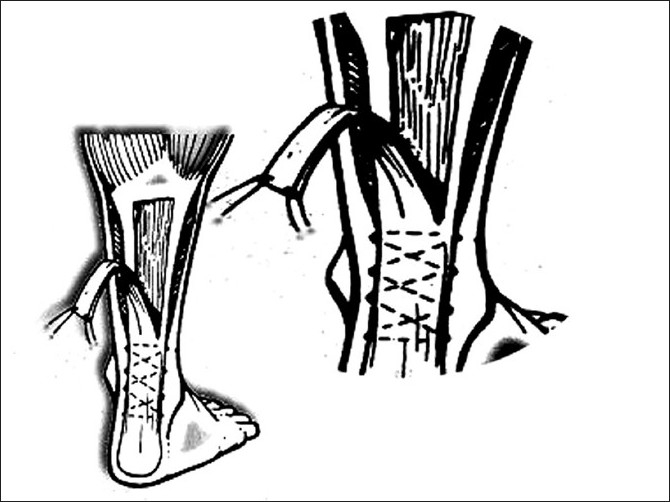
Line diagram showing turn-down of gastrosoleus fascia

**Figure 2:**
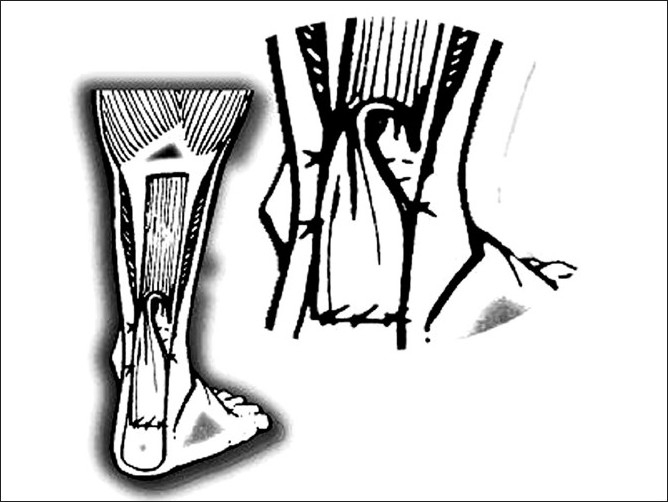
Line diagram showing augmentation of repair with gastrosoleus flap

**Figure 3:**
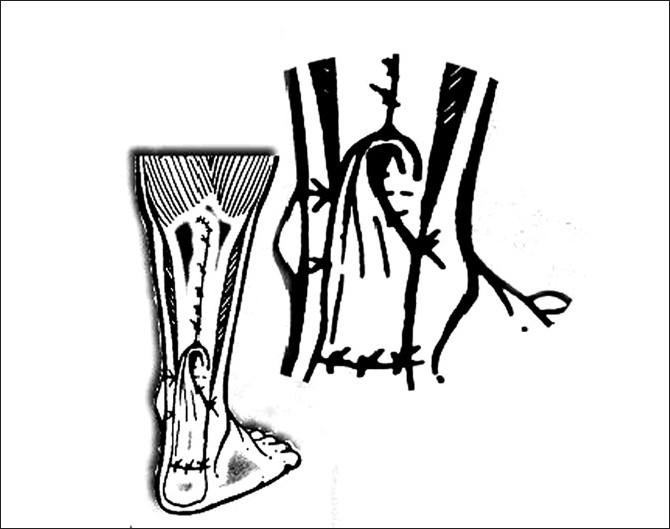
Line diagram showing closure of flap donor site

**Figure 4:**
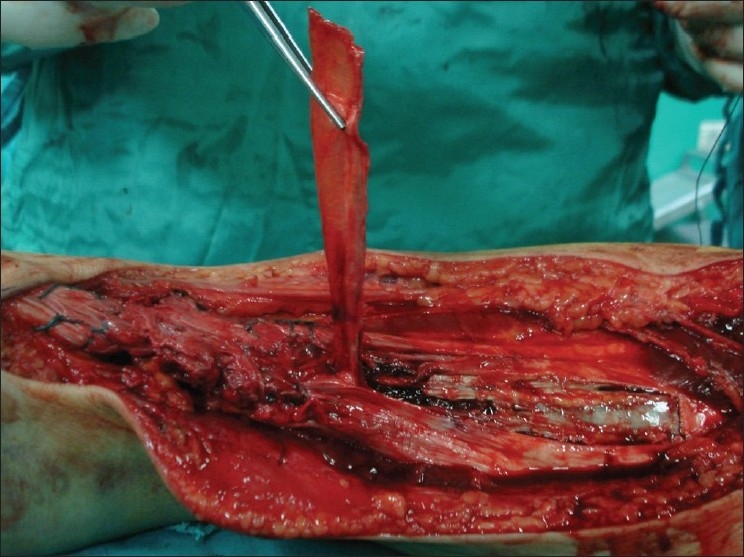
Peroperative clinical photograph showing lifting of gastrosoleus fascia

The repair was then reinforced with a 10–12-cm-long and 2–3-cm-wide strip of gastrosoleus fascia that was twisted 180° on its distal pedicle so that its smooth surface underlies the tendon sheath and the subcutaneous tissues. The defect in the gastrosoleus fascia and the Achilles tendon sheath were repaired with No. 2–0 Vicryl before the closure of the wound [Figures 1–4]. Tourniquet was deflated before wound closure in order to control bleeding and hemostasis. Fascial and skin closures were routine. After surgery, a thin dressing with a sheet of Bactigrass^®^ was applied and the limbs were placed in a short-leg cast by hard 3M Scotch Cast^®^ within the ankle in the neutral position.

The mean operative time was 67 minutes (range 43–106 minutes) and the mean tourniquet time was 62 minutes (range 33–99 minutes).

### Post-operative care

The wound dress was refreshed by a window over the cast 2 days after the operation. Thromboprophylaxis with weight-adapted low-dose heparin was given for 3 weeks. Cast immobilization was continued for 3 weeks. During the cast application period, weightbearing was allowed immediately after surgery as long as the patients could tolerate it. Although synthetic hard 3M Scotch Cast^®^ was used for casting, a cast boat was used to prevent damage to the cast. Three weeks after the surgery, the cast and the skin suture were removed, the wound was examined and the patients were allowed to increase weightbearing progressively on extremity while beginning active and active-assisted range of motion exercises, swimming and stationary cycling. During that period, the ankle was stabilized with a brace (Aircast^®^ Achilles walker, DJO Incorporated, Vista, CA, USA). At the 6-week isokinetic strengthening, isometric and proprioceptive exercises were applied to the extremity for 3 weeks under control of an experienced physical rehabilitation team. Running on an even surface was not allowed before 10 weeks after the operation. Previous athletic activity was allowed at the sixth month.

### Evaluation

**Figure 5:**
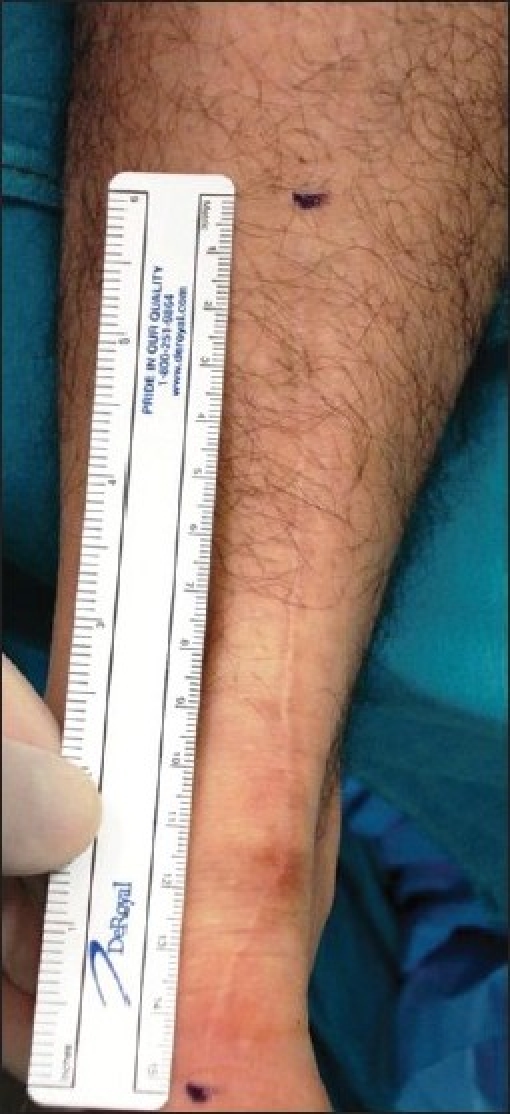
Clinical photograph showing scar of the incision

**Figure 6:**
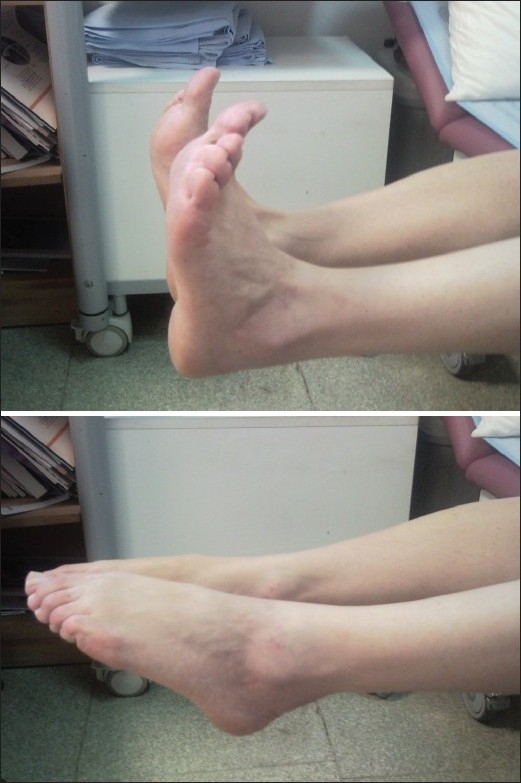
Clinical photograph showing functional result of the operation

All patients were invited to return for an evaluation performed by one of the authors who was not involved in the surgical management of any of cases. At the follow-up, we measured the calf diameter of the injured and uninjured side, examined the ankle range of motion with a goniometer and performed a detailed neurological examination focused on the sural nerve [Figures 5 and 6]. At the last follow-up, visual analogue scale (VAS) was used for pain rating and satisfaction. Questions about the effects of injury and operation to their daily activities/job, condition level of the patients at the time of injury, delayed time for returning to sport and work and any modifications or changes of previous sports activity after treatment was recorded at the last follow-up. As suggested by Kitaoka *et al*., we assessed other factors more specific to repair of an Achilles tendon rupture, namely the strength of ankle plantar flexion with the patient standing on tiptoe, the ability to perform repeated toe raises and single-limb hopping and the neurological status of the foot.^15^ For single-limb hopping, patients were asked to hop as many times as possible until they could not lift the heel off the floor. We noted the complications both from the patients file and the from the follow-up evaluation interview. Also, we used the Rupp score modified by Kerkhoffs *et al*. to evaluate the subjective satisfaction at the most recent follow-up.^16^ Results were rated as excellent (>30 points), good (15–30 points), fair (5–15 points) and poor (<5 points) [Table 2].

**Table 2:** Modified Rupp score for subjective evaluation

Modified Rupp score			
1.	Subjective satisfaction		
	Excellent		5
	Good		1
	Satisfactory		-1
	Poor		-5
2.	Do you experience pain on bearing weight?		
	None		5
	With extended weightbearing		1
	With slight weightbearing		-2
	Continuous pain		-5
3.	Do you experience pain independent of bearing weight?		
	None		5
	Pain associated with changes in weather		1
	Pain sometimes associated with rest		-2
	Continuous pain		-5
4.	Has your ankle function decreased since the operation?		
	No		±2
	Reduction of muscle strength		±2
	Tendency to swelling		±2
	Tendency to cramp		±2
5.	Do you fear rerupture?		
	Yes		-1
	No		1
6.	Do you have limitations in your work?		
	Does not apply		0
	None		5
	Minor		-1
	Major		-3
	Changed profession due to Achilles tendon problem		-5
7.	Do you have limitations in sporting activities?		
	Does not apply		0
	None		5
	Minor		-1
	Major		-3
	Stopped with the activity due to Achilles tendon problem		-5
	Total:
	>30	Excellent	
	15–30	Good	
	5–15	Fair	
	<5	Poor	

### RESULTS

The mean follow-up time was 4.6 years (range 2–9 years). All the patients tolerated the operation well and all of them adapted to the rehabilitation program without any problem.

Table 3 summarizes the comparison of the ankle motion and the calf diameter of the injured and the normal side. The average of ankle plantar flexion was 137° (range, 120–143°) on the repaired side and 139° (range, 120–155°) on the non-injured side. The average of ankle dorsiflexion was 14° (range, 5–21°) on the operated side and 16° (range, 8–22°) on the non-injured side. The mean calf diameter was 39.5 cm (range, 38–43 cm) on the operated side while it was 41 cm (range, 39–44 cm) on the non-injured side. Eight patients experienced transient hypoesthesia in the dermatome of the sural nerve for 6 months after the operation. However, two patients still suffered from sensation loss in this region. At the most recent control, the mean VAS for pain was 0.52 (range, 0–5). Forty-four patients had no pain while 29 had mild pain (VAS score ≤ 4) and five patients still feel uncomfortable (VAS score = 5). None of the patients have severe pain. The mean VAS for patient satisfaction was 8.1 (range, 5–10).

**Table 3:** Comparison of ankle range of motion and calf diameter

	Operated	Non-operated
Plantar flexion (°)	137 (120–143)	139 (120–155)
Dorsal flexion (°)	14 (5–21)	16 (8–22)
Calf diameter (cm)	39.5 (38–43)	41 (34–44)

At the most recent follow-up, 76 patients were able to stand on their tip toes for longer than 30 s. Seventy-five patients could perform repeated toe rises for 30 s. Seventy-one were able to perform single-limb hopping.

All the patients returned to daily activity and none of them had to change or modify their job because of this injury and treatment procedure. Before the injury, 70 patients practiced sports as competitive or recreational on a regular basis. Thirty-six patients defined themselves as well conditioned at the time of injury while 34 were not. Fifty-four patients had returned to their pre-injury sport level without any limitation while 11 had changed their sportive activities such as swimming, jogging or cycling and five of them did not contribute to any sporting activity at all. The mean time to return to previous sports activity was 190.6 (180–228) days.

The rerupture rate was 2.5% (*n*=2) and the average time of rupture after surgery was 16.5 (12–22) months. Both the patients were reoperated with augmentation by fasia lata allograft. The post-operative care was managed by conventional methods. The Rupp scores of these two patients were 3 and 7, and they still feel moderate pain around the injured ankle. Three infections occurred and one of them was deep infection complicated with severe skin necrosis around the incision requiring sharp debridement and plastic reconstruction with rotation flap. This patient had a history of steroid treatment. He returned to previous sport without any limitation after 13 months from the operation and the Rupp score of this patient was 28. Two superficial infections were treated with aggressive wound care and antibiotics. Two patients with diabetes mellitus did not have any problem in wound healing. Deep venous thrombosis developed in one patient after removal of the cast, which was resolved with antithrombotic therapy for 3 months and did not affect the final outcome. Six patients found shoeing uncomfortable because of the prominent hypertrophic scar at the repair site, and these patients used heel cup to overcome this problem.

The mean Rupp score was 29 (3–33) and 92.3% of the patients’ results were considered good or excellent [Table 4].

**Table 4:** Results

Modified Rupp score	%	n
Excellent	62.8	49
Good	29.5	23
Fair	5.1	4
Poor	2.6	2

## DISCUSSION

Rupture of the Achilles tendon can be debilitating and most commonly occurs in 25–40-year-old active individuals. 90% of the patients injured the tendon while playing some form of sport, particularly football. Although age-related risk factors for Achilles tendon injuries are well defined in the English literature, there have been no studies that analyze the relation between the specific form of sports and Achilles tendon injuries.^17^,^18^

The etiology of the Achilles tendon rupture remains unclear, but some of the investigations have supported the theory of chronic degenerative changes based on histological examination of material obtained from the ruptured area during the operation.^19^,^20^ On the other hand, Inglis and Sculo have performed histological examination of acute Achilles tendon rupture and have found evidence of acute pathological changes like hemorrhage and inflammation rather than chronic tendonitis.^5^ In our group, all the 78 patients did not have any kind of prodromal symptoms related with tendonitis before injury. Thirty-nine patients injured their Achilles tendon while playing football on a synthetic grass field. Although most of the Achilles tendon ruptures occur while sportive activities on synthetic field, the published reports about the relation between the Achilles tendon rupture and the synthetic grounds are very limited.^21^,^22^ Also, more than 60% of the patients described themselves non-conditioned and began sportive activity without an appropriate stretching exercise when the tendon rupture occurred. Inglis and Sculco proposed that there is an inhibitor mechanism that regulates the length versus power mechanism of the musculotendinosus unit, which limits the tendon length when sudden overloading of the tendon is applied. They suggest that this mechanism is suppressed or activated by physical condition.^5^ The etiology of the ruptures in these non-conditioned individuals in our series might be related to this inhibitor mechanism.

There are many treatment options for Achilles tendon rupture, such as non-operative closed methods, open surgical repair or percutaneous sutures, which have long been a matter of controversy. The disadvantages of closed procedures are high rerupture rate of 10–30% and less strength and endurance compared with open surgical repair.^23^–^25^ The proper indications for surgical repair appear to be an active patient who demands to return to functional status at the earliest day with a short rehabilitation program. Meanwhile, the operation techniques have been well progressed and the complications of open repair have become less frequent. However, Nistor found only minor differences between the results of surgical and non-surgical treatment.^23^ Simple end-to-end suture is easier to perform and requires a less-extensive dissection, but to approximate a poor quality tendon with only end-to-end suture is not safe. Also, less-invasive techniques have been developed to perform end-to-end suture of the Achilles tendon percutaneously.^10^,^26^ The incidence of sural nerve injury and rerupture rate seems to be higher with these popular techniques.^27^

Augmented repairs provide stronger reconstruction and give more biomechanical stability to the repair. Central gastrosoleus aponeurosis flap repair is superior to standard Kessler repair by virtue of its strength. Gerdes *et al*. have shown, in a series of 18 paired fresh anatomic Achilles tendon, that one flap augmented repair with No. 1 Ticron had an average strength of 217 Newton, whereas conventional repair with two interrupted Kessler sutures (No. 1 Ticron) failed at an average of 154 Newton.^28^ Augmentation by adding collagen to the repair site allows earlier mobility, weightbearing and a more aggressive rehabilitation program with reduction in the incidence of rerupture for both acute and neglected Achilles tendon rupture.^29^,^30^ Nevertheless, Nyyssönen *et al*. retrospectively compared 98 patients of acute Achilles tendon rupture with 59 augmented reconstructions to 39 end-to-end suture and reported that there were no differences in the final outcome. The weakness of this study was that the authors did not seem to consider the strength of the augmented tendon and applied similar rehabilitation program to both groups.^31^

In the recent past, regardless of the treatment methods – non-operative closed methods, open surgical methods or percutaneous procedures – casting in equinus without weightbearing for a minimum of 6–8 weeks has been widely accepted. The current approach to the rehabilitation of Achilles tendon rupture surgery is based on a short period of immobilization and immediate weightbearing because of the complications due to prolonged immobilization, such as arthrofibrosis, joint stiffness, calf atrophy, damage of the articular cartilage and deep vein thrombosis.^32^,^33^ Also, prolonged immobilization and limitation of weightbearing accompanied with prolonged rehabilitation program and sick leave time and a weakened, atrophic, less-vascularized tendon that is prone to reruptures. Although the positive effects of early mobilization on the healing process after surgical repair have been well documented by several reports,^34^,^35^ in these studies full-weightbearing was not allowed for 4–6 weeks. There have been a few animal studies on the effects of collagen response to loading.^28^,^36^ Tensile loads cause viscoelastic response at the muscle–tendon unit and the ruptures are most commonly seen at the musculotendinous junction. Early full-weightbearing after surgical repair by protection from overloading may allow newly formed collagen fiber to grow and be remodeled rapidly, ultimately sustaining tendon strength.^37^,^38^ In the majority of the published series, the results of the primary repair have been reported about Achilles tendon rupture surgery rehabilitated with early weightbearing rather than augmented reconstruction. In our series, the clinical results of augmented repair of Achilles tendon with early weightbearing in walking cast demonstrated satisfactory functional recovery without any increase in the rate of major complications.

Nevertheless, augmented repair of the Achilles tendon rupture has certain handicaps. The major disadvantages of augmented reconstruction are increased rate of wound complication and infection due to the more extensive approach.^39^ A deep infection after surgical repair of an Achilles tendon rupture is a relatively rare but devastating problem as the skin and soft-tissue defects the around ankle are a major challenge for the surgeon. Deep infection and skin loss occurred in one of our patients and was managed with sharp debridement and rotational flap. Methods for the reconstruction of soft tissues after infection have been presented in the literature, but the results have been variable and there is insufficient data in general to support any of the techniques.^40^,^41^ Another problem is enlargement of the posterior site of the ankle. The repaired part of the tendon heals with a bulky tissue that might cause contact ulcers with shoeing. Six patients in our series with uncomfortable shoeing used heel cup to overcome this problem. Although it is not evaluated in the Rupp score, particularly young women patients in our series found the procedure unaesthetic.

Zell and Santoro reported no rerupture in their augmented repair series of 25 acute Achilles tendon rupture, but we had two cases in our series.^30^ Although it was reported that reruptures mostly result from full-weightbearing during the first few days after removal of the cast, orthosis or wrap,^32^,^42^ we did not encounter any immediate rerupture after removal of the cast in our series. However, rerupture is a troublesome complication that is difficult to manage for both the surgeon and the patient. The sural nerve injury can also overshadow the success of the operation even if most of the injuries are transient.^30^,^39^ In order to prevent this complication, the sural nerve can be protected in the lateral skin flap of the posteromedially placed skin incision. In our series, we observed eight patients with sural nerve injuries. We assume that this is because of unnecessary excessive traction, dissection or anatomical variations of sural nerve.

This study includes recreational and competitive athletes, but none of the patients’ was a professional sportsman, and this might be a limitation of this study. We believe that expectations of professional athletes from surgical treatment are completely different from non-professionals. However, all of the patients in this study demanded return to a particular level of pre-injury activity at the earliest date. Surprisingly, in spite of having normal physical examination at the last follow-up, five patients did not return to sportive activity. Garabito *et al*. reported similar findings and they have attributed this result to fear to a new injury, lack of leisure time and the limited number of professional athletes in their series.^39^

A recent article by Metz *et al*. suggests that non-operative treatment of acute Achilles tendon ruptures with functional bracing reduces the number of complications compared with surgical treatment of minimally invasive technique. The difference between treatments for return to sports, pain and treatment satisfaction did not reach statistical significance. Although they concluded that there is a clinically important difference in the risk of complications between treatment protocols, this was not statistically significant.^43^

In conclusion, augmented repair of acute Achilles tendon ruptures using gastrosoleus fascial flaps are strong and stable enough to allow early weightbearing ambulation with favorable clinical results in most of the patients. The disadvantages of the procedure have to be shared in detail with patients before the operation. Care must be taken about wound problems and deep infection because of more extensive dissection. Further studies that include a higher number of professional athletes need to be performed to elucidate the security of this augmented technique.
